# Benign Ancient Schwannoma of the abdominal wall: An unwanted birthday present

**DOI:** 10.1186/1471-2482-10-1

**Published:** 2010-01-06

**Authors:** Ravi K Bhatia, Ayan Banerjea, Manisha Ram, Bryony E Lovett

**Affiliations:** 1 General Surgical Department, Basildon and Thurrock University Hospital Trust, Room B208, Nethermayne, Essex, SS16 5NL, UK; 2 Pathology Department, Basildon and Thurrock University Hospital Trust, Nethermayne, Essex, SS16 5NL, UK

## Abstract

**Background:**

There has been a recent growth in the use of whole body Computerised Tomography (CT) scans in the private sector as a screening test for asymptomatic disease. This is despite scant evidence to show any positive effect on morbidity or mortality. There has been concern raised over the possible harms of the test in terms of radiation exposure as well as the risk and anxiety of further investigation and treatment for the large numbers of benign lesions identified.

**Case Presentation:**

A healthy 64 year old lady received a privately funded whole body CT scan for her birthday which revealed an incidental mass in the right iliac fossa. This was investigated with further imaging and colonoscopy and as confident diagnosis could not be made, eventually excised. Histology demonstrated this to be a benign ancient schwannoma and we believe this to be the first reported case of an abdominal wall schwannoma in the English literature

**Conclusions:**

Ancient schwannomas are rare tumours of the peripheral nerve sheaths more usually found in the head, neck and flexor surfaces of extremities. They are a subtype of classical schwannomas with a predominance of degenerative changes. Our case highlights the pitfalls of such screening tests in demonstrating benign disease and subjecting patients to what turns out to be unnecessary invasive investigation and treatment. It provides evidence as to the consequences of the large number of false positive results that are created by blind CT scanning of asymptomatic patients i.e. its tendency to detect pseudodiesease rather than affect survival rates. Should the number of scans increase there may be an unnecessary burden on NHS resources due to the large numbers of benign lesions picked up, that are then referred for further investigation.

## Background

In recent years private entrepreneurs have recognised, some might say exploited, a market for radiological screening tests [1,2]. One of these tests, Computerised Tomography (CT), in particular whole body CT, is tendered as a possible tool to identify asymptomatic thoracic and abdominal disease. However, before any procedure is adopted to screen for disease, its cost, benefit and risk should all be taken into account and before this happens the value of the test must remain unproven and unknown[3] To date, this evaluation has not been carried out with whole body CT screening in asymptomatic individuals [4]. This may be due to the huge cost and long follow up period required for such a trial as well as the fact that grants are unlikely to be given to research what is increasingly seen as an entrepreneurial scheme [2]. Hence, a number of clinical, ethical and health-economical issues have been raised by medical professionals in response to the growth of such an unproven test in the private sector. Although these centre on the lack of evidence that blind CT scanning of asymptomatic individuals produces any benefit in terms of identifying or precluding disease, they also emphasise the potential harm from radiation as well as from further, often invasive, investigations which are offered in the large number of false positive cases frequently seen. Furthermore, some have voiced concern at the growing public expenditure spent on investigating the large number incidental lesions uncovered on private CT scans. In this paper, we present our treatment for a lesion identified on a private whole body CT scan, given to our patient as a birthday present, as well as recount what we believe to be the first reported case in the English literature of an abdominal wall ancient schwannoma. We discuss the clinical, histological and radiological features of ancient schwannomas, as well as discuss in further depth, the controversy surrounding whole body CT screening in asymptomatic individuals.

## Case Report

A healthy 64 year old lady received a LifeScan (privately funded whole body CT scan) for her birthday which revealed an incidental mass in the right iliac fossa (RIF). This was investigated further with a colonoscopy which revealed small benign polyps only. Initially a putative diagnosis of GastroIntestinal Stromal Tumour (GIST) was considered and the patient referred to our NHS Colorectal outpatient clinic.

**Figure 1:**
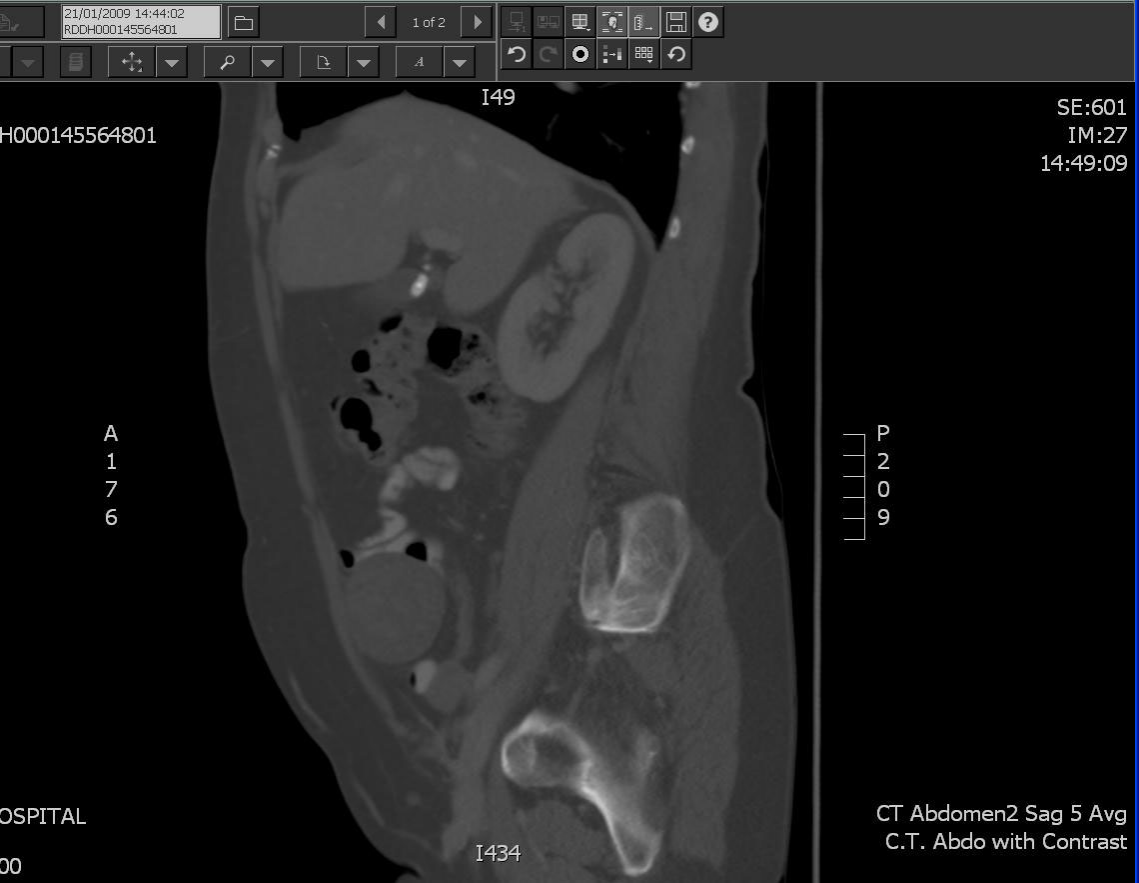
**Saggital section of CT Abdomen with Contrast**. Well circumscribed RIF mass next to anterior abdominal wall.

Clinically, the lady was well with no weight loss, anaemia, change in bowel habit or loss of appetite. She complained of RIF discomfort but admitted that this had only manifested since her CT scan. She was extremely anxious about her condition and the underlying diagnosis. On examination, a non-tender 8 cm mass was palpable in the RIF and a routine blood screen was entirely normal. The case was discussed at the Lower Gastrointestinal Multidisciplinary Team (MDT) meeting and it was agreed that a repeat CT scan with contrast should be performed. The scan demonstrated a 6 × 5 cm heterogeneous mass in the RIF adjacent to the peritoneal wall (Figure 1). No other abnormality was evident but a normal appendix could not be identified. These findings were discussed again at the MDT meeting and a differential diagnosis of mucinous tumour or chronic mucocele of the appendix was suggested.

**Figure 2:**
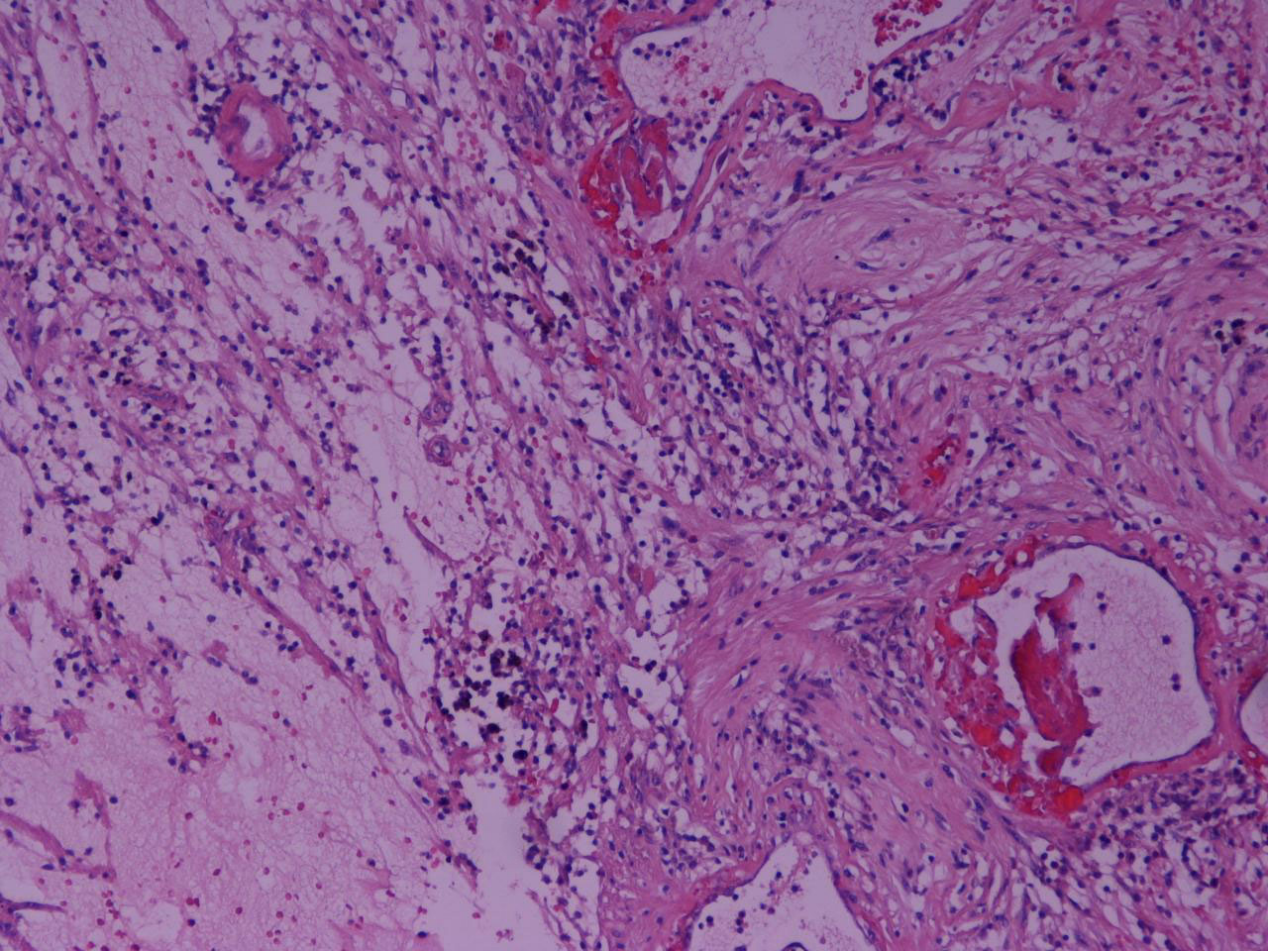
**Microscopic appearance of the lesion**. Pronounced degenerative changes including myxoid stroma, haemorrhage, conspicuous blood vessels with hyaline thickening of walls and luminal thrombosis (H&E ×20)

**Figure 3:**
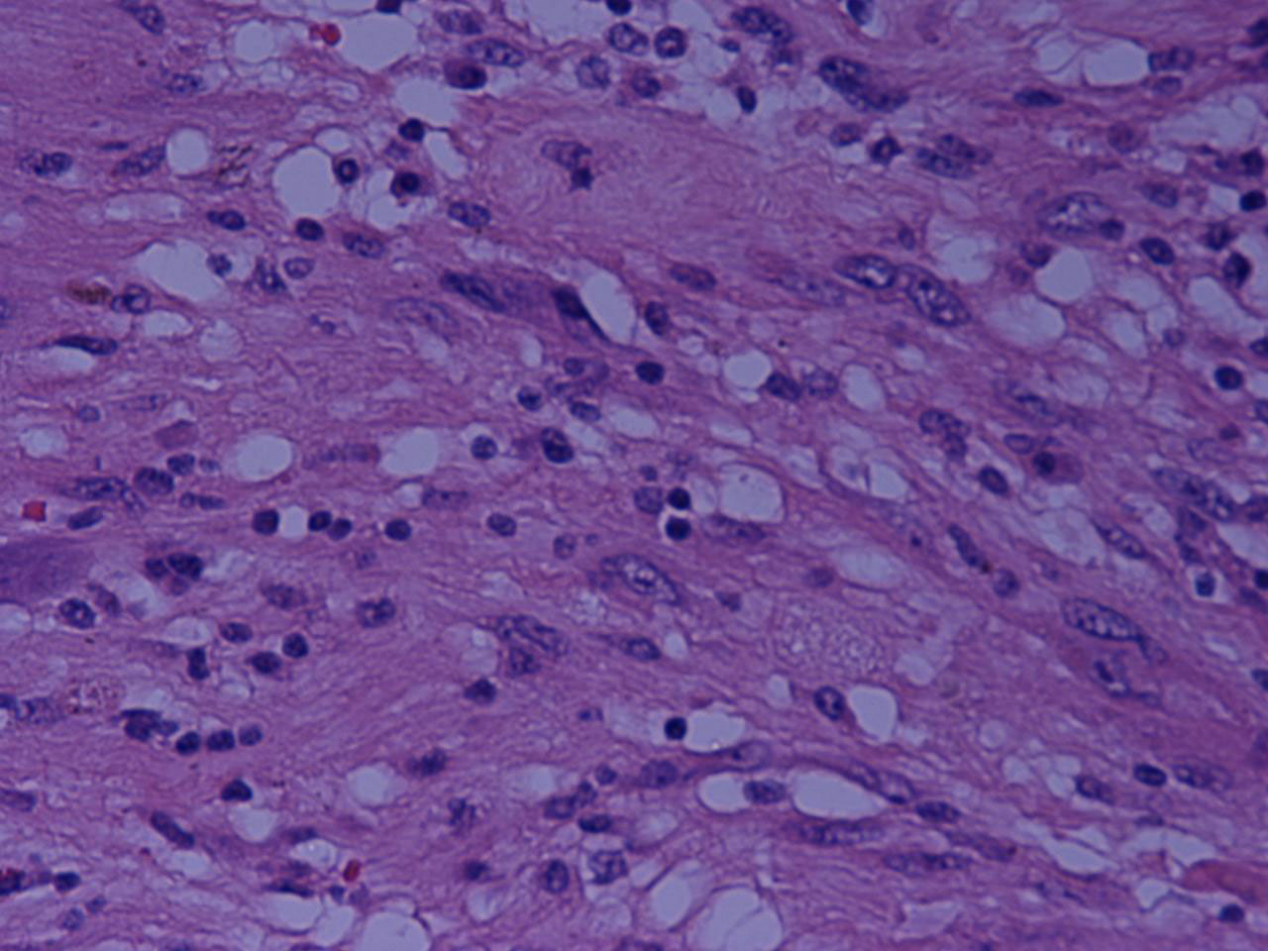
**Microscopic appearance of the lesion **Focal marked nuclear pleomorphism, with isolated cells having bizarre hyperchromatic nuclei (H&E ×60)

The possible diagnoses were discussed with the patient and after consideration of the risks and benefits, she agreed to surgery by a laparoscopic approach. At laparoscopy a 6 × 4 × 3.5 cm extraperitoneal ovoid nodule was found on the abdominal wall in the RIF. In light of the unusual appearances, the procedure was converted to laparotomy but no intra-peritoneal pathology was noted. The lesion was excised and sent for histological examination. Macroscopically, it had a solid cut surface with tan, brown and cream areas and contained a 1.2 cm cyst. Microscopically, it was composed of hypocellular and hypercellular areas. The hypocellular areas were oedematous and myxoid, containing chronic inflammatory cells and spindle cells which were haphazardly arranged, aggregating in areas to form small focal palisades. The spindle cells showed hyperchromatic and irregular nuclei although no mitotic figures were seen (Figures 2 and 3). There were several foci of stromal hyalinisation and sclerosis, with numerous ectatic blood vessels. Immunohistochemistry showed that the spindle cells were strongly positive for S100 protein and a final diagnosis of benign ancient schwannoma was made. Postoperatively, the patient recovered well and was discharged 2 days after surgery. She is now asymptomatic.

## Conclusions

Ancient schwannomas are rare tumours originating from Schwann cells in the peripheral nerve sheath [5]. Up to 20% of cases are associated with Neurofibromatosis Type 1 [6]. They usually arise in females [7] between the ages of 20-50 years, [6] with a predilection for the head, neck and flexor surfaces of extremities [8,9]. However, there have been sporadic cases of these tumours arising in the retroperitoneum, pelvis, perineum, adrenals, kidneys and even masquerading as an inguinal hernia [9-14]. They may be asymptomatic and only found incidentally on examination or imaging. However, occasionally they produce pressure effects on surrounding large nerves [15].

Ancient schwannomas are a subtype of classic schwannomas with a predominance of degenerative changes including cyst formation, calcification, haemosiderin deposition, interstitial fibrosis, and vascular hyaline degeneration [16]. They show spindle cells with focal nuclear palisading patterns [6] arranged in distinctive dense (Antoni A) and loose (Antoni B) areas [6,16]. Usually, connective tissue fragments (Verocay bodies) and intranuclear vacuoles (lochkern) are present [17-19]. These features may sometimes be seen on CT, which may demonstrate a well circumscribed, heterogeneous mass of low density which fits with the microscopic pattern of cellular and hypocellular areas. The term "ancient" was used as a description for the degenerative changes apparent on microscopy [20,21] which was thought to be due to the increasing tumour size causing vascular insufficiency [22].

There have been less than ten reports in the literature of malignant lesions arising from schwannomas [23-25] although some argue that these malignant tumours may arise *de novo *[7] rather than having undergone secondary anaplastic change [19,26] and that recurrence after resection may actually be due to incomplete excision [27].

Schwannomas react strongly with S100 protein and immunohistochemistry can be used to aid diagnosis and to differentiate them from malignant peripheral nerve sheath tumours [24,28,29]. This is especially helpful as the occasional appearance of hyperchromatic cells and cytological atypia may lead to diagnostic difficulty [19,22] especially when fine needle aspiration cytology (FNAc) is used to make a diagnosis [5,7,18,21,22,28,30,31] as this technique rarely produces an adequate enough sample [29]. False positive diagnosis of malignancy may lead to over treatment with radical resections being carried out unnecessarily. Together with the clinical history, the characteristic radiological findings may help distinguish schwannomas and prevent unnecessary resection for such a benign condition [32-34]. However, as in our case, this is rarely possible and surgical resection is still common [6,27,35,36].

Our case highlights one of the main pitfalls of whole body CT scan used as a screening method for disease. This lady suffered considerable mental anguish, endured extremely invasive procedures and the significant risks that accompany them, for a condition that may never have caused any symptoms. Studies have shown that up to 86% patients who undergo these scans show at least one abnormal finding and up to 37% of individuals receive recommendations for further investigation [37]. Whether these findings translate into clinical significance is doubtful. Given the large number of findings identified by such a crude test, there have been calls for guidelines to be drawn up to as to help clinicians decide which abnormalities warrant further investigation [38,39]. Although such guidelines may be helpful, this may be harder for lesions detected on abdominal or pelvic CT given that the often non contrast CT method used for screening, rarely provides enough detailed information on which to guide management. Additionally, different guidelines would have to be drawn up for different organs. Yet this would have been of little benefit in our case as the lesion was adherent to the anterior abdominal wall. In spite of this, the few supporters of screening CT do point out that as with mammograms, false positive results may decrease with increased use and experience of the test [40].

As well as the high false positive rate, the other main criticism levelled is that there is scant evidence that whole body CT reduces morbidity or mortality [2] even if it were to correctly identify true positives or negatives. For example, lead time bias may mean earlier diagnosis of disease but not improved survival. Length bias may mean overrepresented diagnoses of slower progressing disease (more likely to be picked up by a static screening test such as a CT scan) but again not actually improved survival times. Overdiagnosis bias (demonstrated effectively in our case) occurs when pseudodiesease- i.e. cases that probably wouldn't have caused disease were they not picked up, is not adjusted for [41]. True negative results would of course reassure individuals, however this may lead them carrying on with unhealthy lifestyle habits in the knowledge that they have been given the "all clear". Of course, false negatives are probably the worst outcomes for both the patient and reporting radiologist [2].

Given the lack of evidence of any tangible benefit of CT screening tests, some have speculated they may actually do more harm than good. There is an ongoing debate as to whether the risks of radiation from whole body CT may be harmful to asymptomatic individuals and differences stem from the calculation of radiation dose depending on type of scanner and protocol used or whether interval CT scans are offered [42,43]. Although it is hard to prove these associations, given that we are cautious with regards to radiation exposure in our usual practice it seems prudent to apply the same hesitancy when it comes to unproven screening tests.

But what for the argument that CT screening tests offer individuals control over their own healthcare for those that can afford it? Although patients who present for these tests may value the consumer empowerment over their healthcare management and desire "comprehensive wellness" (i.e. assurance that they are free of disease even in the absence of symptoms), [44] one has to remember that CT screening may have a large false positive rate and as in our report, the costs of the additional investigations will mostly be borne by the NHS, despite the initial screening test being performed in the private sector. There is concern that should CT screening become more widespread, the financial burden of distinguishing the incidentalomas from true disease be may end up being shared by the taxpayer and thereby increase national health expenditure [1,44] hence widening the disparity of healthcare options for the less well-off [44]. Of course this is an argument that may be made of other unproven treatments performed only in the private sector. However the targeted marketing of CT screening to the asymptomatic, economically comfortable and educated health conscious consumer [45] as well as the prima fasciae logic embedded such a test should the benefits and risks not be adequately explained, brings the potential for a huge number of "worried well" presenting to publicly funded surgical outpatients clinics.

In this study we have presented the first reported case in the English literature of an abdominal wall ancient schwannoma and discussed the key clinical, radiological and pathological features of the condition. As this was picked up asymptomatically on a private health whole body CT screen given to the patient as a birthday present, we have extolled the pitfalls of such tests as well as offered an insight into the clinical, ethical and health economic issues that they raise. Wider marketing and availability of these services may lead to increased non evidence based patient-led screening in the private sector. Unfortunately, this may add an unnecessary burden to public health resources.

## Consent

Written informed consent was obtained from the patient for publication of this case report and any accompanying images. A copy of the written consent is available for review by the Editor-in-Chief of this journal.

## Competing interests

The authors declare that they have no conflicts of interest. The authors alone are responsible for the content and writing of this paper

## Authors' contributions

RKB was the principal author of the paper, carried out a literature review of the topic and produced the original manuscript. AB modified this. MR proof read the document and provided the pathological slides for inclusion into the manuscript. BEL gave final approval for the manuscript to be submitted. All authors read and approved the final manuscript.

## Author information

RKB is a house officer in General Surgery. BEL is a Consultant Surgeon. AB and MR are Specialist Registrars in General Surgery and Pathology respectively.

## Pre-publication history

The pre-publication history for this paper can be accessed here:

http://www.biomedcentral.com/1471-2482/10/1/prepub
